# Rapid genotyping of targeted viral samples using Illumina short-read sequencing data

**DOI:** 10.1371/journal.pone.0274414

**Published:** 2022-09-16

**Authors:** Alex Váradi, Eszter Kaszab, Gábor Kardos, Eszter Prépost, Krisztina Szarka, Levente Laczkó

**Affiliations:** 1 Department of Metagenomics, University of Debrecen, Debrecen, Hungary; 2 Department of Laboratory Medicine, University of Pécs, Pécs, Hungary; 3 Veterinary Medical Research Institute, Budapest, Hungary; 4 ELKH-DE Conservation Biology Research Group, Debrecen, Hungary; University of Helsinki: Helsingin Yliopisto, FINLAND

## Abstract

The most important information about microorganisms might be their accurate genome sequence. Using current Next Generation Sequencing methods, sequencing data can be generated at an unprecedented pace. However, we still lack tools for the automated and accurate reference-based genotyping of viral sequencing reads. This paper presents our pipeline designed to reconstruct the dominant consensus genome of viral samples and analyze their within-host variability. We benchmarked our approach on numerous datasets and showed that the consensus genome of samples could be obtained reliably without further manual data curation. Our pipeline can be a valuable tool for fast identifying viral samples. The pipeline is publicly available on the project’s GitHub page (https://github.com/laczkol/QVG).

## Introduction

The first-hand experience of the severe acute respiratory syndrome coronavirus 2 (SARS-CoV2) pandemic is that effective outbreak management requires fast and strain-level identification of the causative pathogens. The most fundamental information about microorganisms might be their accurately reconstructed genome sequence, which can provide insight into the evolution of pathogens and the clinical outcomes of outbreaks [1]. The application of Next Generation Sequencing (NGS) revolutionized the identification and study of microorganisms by providing an ever-increasing amount of genome sequence data available for data processing and research. Although laboratory instruments are available for numerous research and medical facilities [2], the lack of bioinformatic tools became a bottleneck that hinders high-throughput analysis. Therefore, new, widely, and openly available bioinformatic tools are needed to keep pace with the increasing speed of data generation, and the growing amount of data capable of performing the rapid and accurate analysis of multiple samples sequencing reads.

Although open-source virus genome reconstruction and identification tools exist, some of them are limited or optimized to one species; e.g. HCV [3] is optimized for hepatitis C, MinVar [4] for the HIV-1, and ViralFlow [5] for the SARS-CoV2 virus. These tools may be ideal for genotyping a given viral genome, but their broad applicability may be limited by their species-specific design. Different pipelines focus on different output formats with examples of limited (e.g. although being very user-friendly, the main output of MALVIRUS [6] is a vcf file of variants) and very rich outputs (e.g. the consensus and statistics of TRACESPipe [7] and nfcore-viralrecon [8, 9]. There is also great variability in the utilization of bioinformatic tools in openly available pipelines. TRACESPipe [7] uses bwa [10] or bowtie2 [11], of which the latter can be slower under certain conditions and can show improper pairing of sequence mates [12]. The performance of these aligners and the trade-off between sensitivity and computational time are influenced by sequencing data quality and the setting of software options. TRACESPipe [7] then uses *de novo* assembly to obtain the possible most complete and accurate consensus sequences. The target-based version of the TRACESPipe [7] pipeline, TRACESPipeLite [13], utilizes bwa [10] by default to align the reads to the reference genome. V-pipe [14] utilizes LoFreq [15] or ShoRAH [16], and viralrecon [8, 9] uses iVar [17] (capable of analyzing multiple samples simultaneously) as default for amplicon-based datasets to call polymorphisms, all of which variant callers might show a lower accuracy [18, 19]. The variant caller in viralrecon [8, 9] be changed to bcftools [20] (default for metagenomic datasets), a variant caller with higher accuracy [19]. Freebayes [21] has comparable accuracy to bcftools [22] and, owing to its customizability, may be ideal to adapt to a wide range of datasets. However, to our knowledge, freebayes [21] is rarely applied in pipelines aiming at reconstructing viral diversity, although the constant development of this tool may contribute to its widespread adoption. One exception is ViReflow [23], which relies on the utilization of specific, potentially costly services, such as the Amazon Web Services (AWS) cloud computing resources to achieve a high analysis speed.

This paper presents our approach to the accurate reference-based mass analysis of targeted viral genomes. The pipeline was developed in bash and can be parameterized from the command line. We aimed to combine a rich set of analysis tools for the comprehensive analysis of viral variability of samples. In our work, we automatized the reconstruction of the dominant consensus genome sequence, its’ annotation, and the within-host variability. Our pipeline also outputs statistics of sequencing quality and analyses breadth of coverage and read depth. Input samples can be specified using a list of sample file basenames. Our goal was to make the presented pipeline user-friendly while supporting its adaptation to a wide range of datasets with maintaining accuracy and promoting the quick analysis of samples. We paid attention to avoiding the usage of proprietary software to enhance the availability and transparency of the method. Our pipeline is freely available on the project’s GitHub page (https://github.com/laczkol/QVG).

## Description of the pipeline

**Fig 1:**
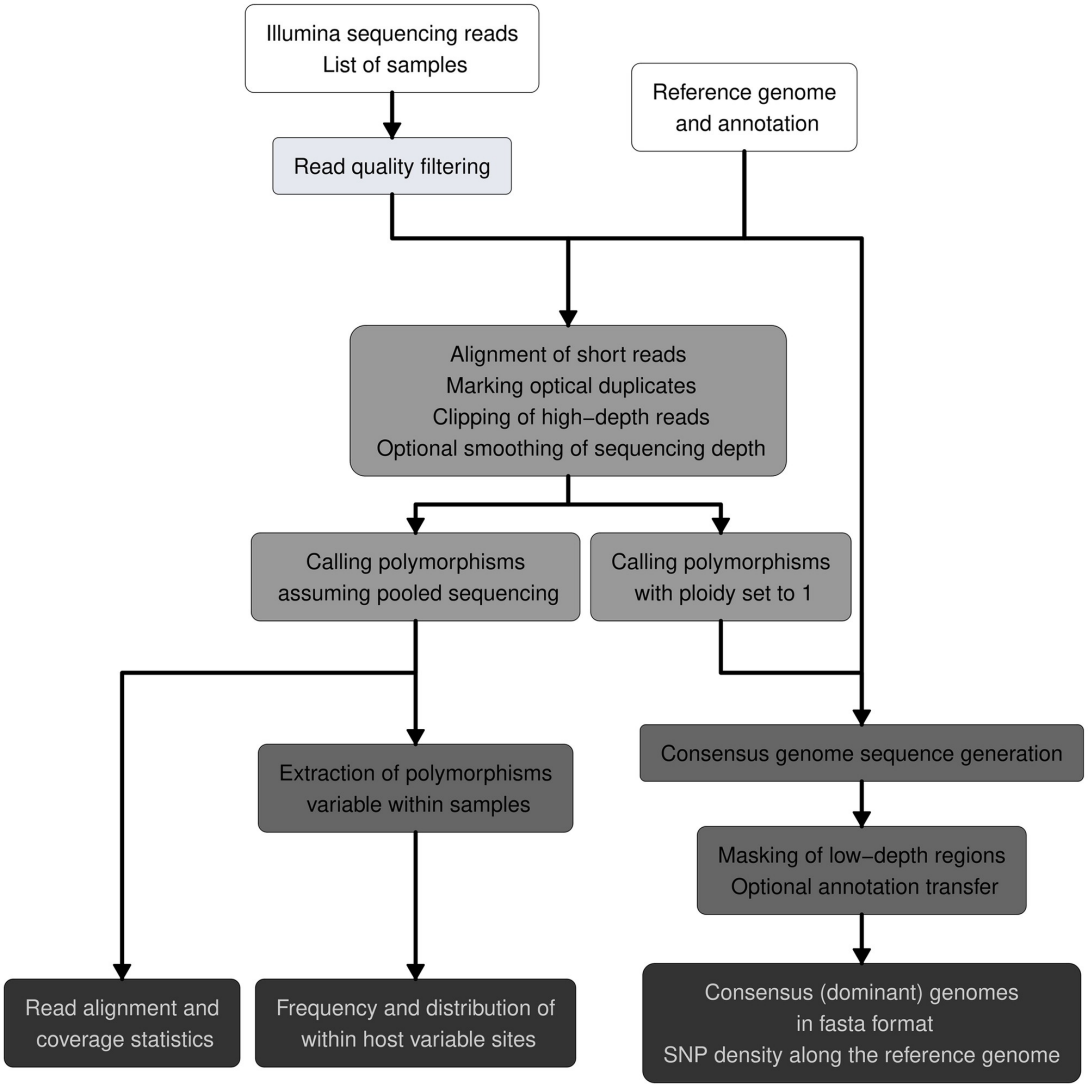
Schematic representation of the QVG pipeline. White boxes represent input data needed to run the pipeline, and differently shaded gray boxes show the main consecutive steps of the workflow proposed in this study. The main outputs are shown in dark gray boxes.

The pipeline relies on existing tools to characterize samples using NGS data and is designed to readily use the output of any Illumina platform in.fastq format. The method (Fig 1) can be applied to both single-end and paired-end sequencing. First, reads are checked for quality and adapter content using fastp 0.20.1 [24], and statistics are exported to.html format. This step is able to trim and quality filter the reads to remove sequencing biases. The sequencing reads are kept separate while maintaining the order of read pairs. Since the alignment specificity tends to decrease with shorter read lengths [12], we do not suggest using reads shorter than 72 base pairs (bp). The filtered reads are aligned to the reference genome sequence using bwa 0.7.17 [10]. Next, duplicates (i.e. PCR duplication artifacts and optical duplicate reads originating from the same DNA fragment incorrectly identified as two separate clusters) are marked with sambamba 0.8.2 [25] and descriptive alignment statistics, including reference genome breadth (i.e. the fraction of the reference genome covered by any number of reads), read depth, samtools’ simple statistics (flagstat) and index statistics, are produced with samtools 1.15.1 [26]. Sample files are subset to include only samples covering at least a given proportion of the reference genome (default is 90%). Statistics are plotted using R 3.5 [27] and summarized in.pdf files. Prior to genotype calling, using bedtools 2.29.2 [28] and sambamba slice 0.8.2 [25], high-depth alignment positions are clipped with a default threshold of 10 times the mean sequencing depth. Additionally, if sequencing depth bias is expected [29] the evenness of the read depth can be improved by resampling the depth using consecutive genomic windows to a fixed number of alignments. If this feature is turned on, the pipeline looks for 500 alignments in 100 bp long genomic windows as default values. This smoothing of reads aims to both decrease the running time of variant calls and the frequency of false polymorphisms. The threshold of clipping and optional resampling can be set using the command line to boost adaptability. Clipping of high-depth alignments is carried out before resampling. To capture the polymorphisms of samples, two variant calls are performed, both of which use freebayes 1.0.0 [21] and parallel [30] to call variant positions of multiple samples simultaneously. We set freebayes to use the five most probable alleles and annotate variants only with a minimum read depth of five. Base quality scores and mapping quality must have a value larger than 30 to include in variant calling. Alternative alleles with a frequency lower than 20% are excluded from this step. We run freebayes with clumping of haplotypes disabled, Hardy-Weingberg Equlibrium (HWE) priors turned off, and use the mapping quality, read placement, strand balance, and read position probability instead. Ploidy is set to one in the first variant call to annotate the dominant genome’s polymorphism. The computationally most intensive step of the pipeline is variant call. The number of samples analyzed simultaneously in this step equals the number of CPU threads specified, utilizing all the memory needed to genotype those samples simultaneously. Using vcflib 1.0 [31] variants are filtered for a minimum quality of 10 and a ratio of quality / alternate allele observation count of 10 (i.e. each observation is required to have a quality score of at least 10) to remove poor quality variants discovered on alignments with low mapping quality due to, for example, aligning the reads to repetitive regions. This filtering aims to decrease the frequency of false positive polymorphisms, potentially distorting the results of downstream analyses relying on genomic variability. Then, single-nucleotide polymorphism (SNP) density in 1kbp consecutive windows is extracted from the resulting.vcf files is extracted using vcftools 0.1.16 [32] and visualized using R 3.5 [27]. Vcf statistics as exported by vcfstats 1.0 [31] in plain text files within the output directory. The sequence of the dominant genome is retrieved using vcf2fasta [31]. The filtered reads are aligned to this resulting.fasta file, and regions with a read depth lower than the minimum read depth set for variant calling are masked out with ’N’-s using bedtools 2.92.2 [28].

As *de novo* mutations and/or multiple acquisition sources might introduce genetic heterogeneity of samples [2, 33], a second variant calling step is performed with ploidy unset and assuming pooled sequencing. Low-frequency variants resulting from sequencing error, like in the first variant call, are filtered out [34]. This step aims to give insight into the population diversity described by allele balance (AB) after filtering the abovementioned variants. The genotypes with their corresponding AB are saved to plain text files using bcftools 1.9 [20] and visualized using R 3.5 [27].

This way, running the pipeline exports the dominant viral genome of samples and provides insight into the intra-host diversity. With GNU parallel [30], tasks are run in parallel to decrease computation time. Using Liftoff 1.6.3 [35], annotations of the reference genome (in gff3 format) can be transferred to the consensus sequences output by QVG.

### Benchmarking

The pipeline presented in this study was tested on different operating systems, namely, Ubuntu Server 20.04, Linux Mint 20.2, Debian 10.1, and 11.0. However, it can run using any UNIX-like operation system with dependencies installed correctly. Requirements of the pipeline were installed using the conda package manager as specified in the yaml configuration file uploaded to the github repository of the pipeline (https://github.com/laczkol/QVG).

The accuracy of the pipeline was tested using synthetic datasets. We simulated sequencing reads based on the sequence of six viral genomes that were also used as references for the validation on real data. First, we introduced mutations in the sequences using Mutation-Simulator 3.0.1 [36] with a SNP-rate of 0.05. Then, using wgsim 1.10 [37], we simulated 150 bp long paired-end sequencing with coverage values (i.e. the number of times the sequenced nucleotides cover the reference genome) of 100×, 1000×, 5000×, and 10000× as implemented in readSimulator 0.01 [38]. We set the error rate to 0.1%, which can be commonly observed in the middle of the reads [39]. We run QVG by specifying the original (i.e. non-mutated) genome as the reference sequence. Next, we compared the.vcf file output by QVG with the known mutations introduced by Mutation-Simulator and calculated sensitivity (true positive rate—TPR), specificity (true negative rate—TNR), balanced accuracy (BA), and precision (positive predictive value—PPV) using the following formulas:

$$TPR=\frac{number\;of\;true\;positives\left(TP\right)}{number\;of\;true\;positives\left(TP\right)+number\;of\;false\;negatives\left(FN\right)}$$

$$TNR=\frac{number\;of\;true\;negatives\left(TN\right)}{number\;of\;true\;negatives\left(TN\right)+number\;of\;false\;positives\left(FP\right)}$$

$$BA=\frac{TPR+TNR}{2}$$

$$PPV=\frac{number\;of\;true\;positives\left(TP\right)}{number\;of\;true\;positives\left(TP\right)+number\;of\;false\;positives\left(FP\right)}$$

We defined the number of true positives (TP) as the number of known mutations found by the pipeline, whereas the number of false negatives (FN) was measured as the number of known mutations that were not identified by QVG. The number of false positives (FP) showed the number of mutations identified as polymorphic positions after genotyping, which were not mutated before read simulation. The number of true negatives (TN) constituted sites that were not mutated by Mutation-Simulator and were neither identified as polymorphic by QVG. We visualized the results using the ggplot2 [40] R package [27].

The performance of the pipeline was validated on multiple real datasets described below. In the first run, samples of the given dataset (Table 1) were analyzed simultaneously using six CPU cores; then, to assess the correlation of running time, read depth, and the number of reads supplied for the run, samples were genotyped one by one using one CPU core.

**Table 1:** Summary of datasets used in this study.

Dataset	Sequencing method	NCBI SRA accessions	Reference genome size (bp)	Genome sequencing approach	Reference
SARS-CoV2 (this study)	MiSeq PE 150 bp	SRR19666963, SRR19666962, SRR19666951, SRR19666950, SRR19666949, SRR19666948, SRR19666947, SRR19666946, SRR19666945, SRR19666944, SRR19666961, SRR19666960, SRR19666959, SRR19666958, SRR19666957, SRR19666956, SRR19666955, SRR19666954, SRR19666953, SRR19666952	29,903	Amplicon-based	This study
SARS-CoV2 (public)	NovaSeq PE 150 bp	SRR14824570, SRR17309642, SRR16741159, SRR14155371, SRR16912480, SRR14824567, SRR14824569, SRR14824574, SRR14824563, SRR14155385, SRR14824566, SRR14824573, SRR14824560, SRR14824572, SRR14824562, SRR14824561, SRR14824565, SRR16912539, SRR14824564, SRR14824568	29,903	Amplicon-based and genomic	INSDC SARS-CoV-2 Viral Sequencing Data
Hepatitis B (HBV)	MiSeq PE 150 bp	SRR12535936, SRR12535937, SRR12535938, SRR12535946, SRR12535947	3,182	Amplicon-based	Hebeler-Barbosa et al., 2020 [41]
Rabies (RABV)	HiSeq PE 125bp	SRR12012243, SRR12012256, SRR12012246, SRR12012251, SRR12012238, SRR12012242, SRR12012241, SRR12012234, SRR12012239, SRR12012247, SRR12012255, SRR12012245, SRR12012236, SRR12012253, SRR12012240, SRR12012237, SRR12012244, SRR12012250, SRR12012252, SRR12012254, SRR12012248, SRR12012249, SRR12012235	11,923	Genomic	Sabeta et al., 2020 [44]
Avian adenovirus	NextSeq SE 150 bp	N.A.*	45,473	Genomic	Homonnay et al., 2021 [46]
Feline coronavirus (FCoV)	MiniSeq PE 150 bp	SRR8352624	29,174	Genomic	de Barros et al., 2021 [45]
Herpes Simplex Virus 1 (HSV-1)	MiSeq PE 250 bp	ERR3316622, ERR3316623, ERR3316627, ERR3316619	152,222	Genomic	Lassalle et al., 2020 [49]

*Raw Illumina reads were kindly made available for us by Homonnay et al. (2021) [46] upon request.

We tested the performance of our pipeline by comparing the results obtained by Quick Viral Genome Genotyper (QVG) against the output of Geneious Prime 2021.2.2. Owing to its ease of use, Geneious is one of the most widely used cross-platform commercial software to carry out reference-based genotyping of samples. For this comparison, we used 20 SARS-CoV-2 positive nasopharyngeal samples (Table 1) (New Coronavirus Nucleic Acid Detection Kit (Perkin Elmer, Waltham, MA, USA); samples with <30 threshold cycle were chosen) to sequence the virus genome. RNA was extracted using the Viral DNA/RNA extraction kit and Automated Nucleic Acid Extraction System-32 (BioTeke Corporation, Beijing, China) then libraries were prepared with NEXTFLEX^®^ Variant-Seq^™^ SARS-CoV-2 Kit (For Illumina^®^ Platforms) (Perkin Elmer, Waltham, MA, USA). The libraries were processed in an Illumina MiSeq platform using a MiSeq Reagent Kit v3 (Illumina, San Diego, CA, USA) following the manufacturers’ instructions. As a reference sequence for this experiment, we used the genome of the SARS-CoV2 isolate Wuhan-Hu-1 (MN908947.3). In Geneious, after removing duplicate reads, reads were mapped to the reference genome using the Geneious mapper with the default sensitivity (Medium Sensitivity/Fast). Before mapping, sequences were trimmed the same way as in the QVG pipeline. As a final step of genotyping using Geneious, we visually inspected the alignments of reads and manually corrected ambiguous sites and obvious genotyping errors by substituting such sites with the highest frequency nucleotide. This procedure took ~10–15 minutes per sample. Geneious was run on a computer with an Intel Core i7-11700K 3.60GHz CPU running Windows 10 64-bit. Using the parameters described above, the genome sequences obtained by both approaches were submitted to the Pangolin web server (https://cov-lineages.org/resources/pangolin.html) to assign each sample to its corresponding lineage.

In addition, we collected and re-analyzed publicly available SARS-CoV-2 sequencing reads with known identity (Table 1). These raw sequencing data were either produced by amplicon-based sequencing (lineages Alpha, Beta, Gamma, Epsilon, Eta) or a transcriptomic sequencing approach (lineage Omicron). Publicly available samples were genotyped, relying on the same reference genome and parameter values we used for our newly generated sequencing data. Re-analyzed consensus genome sequences were submitted to the Pangolin webserver (https://cov-lineages.org/resources/pangolin.html), then we compared the assigned lineage to the originally reported one (see Table 1 for accession numbers).

Another amplicon sequencing-based dataset used for the benchmarking was the dataset presented by Hebeler-Barbosa et al. (2020) [41]. Raw reads of 5 Hepatitis B (HBV) virus samples were supplied to our pipeline. Samples were genotyped using the read alignment to the reference genome of the Hepatitis B virus (strain ayw) (NC_003977). The consensus genome sequences were submitted to the Genome Detective’s HBV phylogenetic typing tool (https://www.genomedetective.com/app/typingtool/hbv/ [42]). This tool not only reports the most probable lineage assigned to samples but conducts a recombination analysis using bootscan [43]. We compared the genotypes assigned by Genome Detective with the originally reported lineage by Hebeler-Barbosa et al. (2020) [41].

To demonstrate that our approach can process not only AmpliSeq datasets, we run the Rabies virus (RABV) dataset presented by Sabeta et al. (2020) [44] through our pipeline. Sequencing reads of this dataset were obtained after the depletion of host DNA and RNA [44]. To genotype the samples of this dataset, we used the genome of Rabies virus (isolate 20034) (KT336433). We checked the identity of samples by submitting the consensus genome sequences to the RABV-GLUE identification tool (http://rabv-glue.cvr.gla.ac.uk/), then compared the most probable lineage uncovered by this tool with the identity of the originally reported lineage. Furthermore, we re-analyzed the feline coronavirus (FCoV) sequence data of de Barros et al. (2019) [45], and the avian adenovirus sequencing reads of Homonnay et al. (2021) [46], the latter of which was the only single-end read sequencing dataset included in the benchmarking. For the FCoV dataset, we reduced the minimum read depth required for variant calling to three, as this sample showed the lowest mean read depth after aligning the reads to the reference genome of feline coronavirus (isolate UG-FH8) (KX722529) also used by de Barros et al. (2019) [45]. The genotyping of the adenovirus sample used the reference genome of the fowl aviadenovirus B strain (40440-M/2015) (MG953201). Since no subtyping tool exists for the latter two viral species, we used blastn to match the consensus genome sequence against the NCBI nucleotide collection database; then, the retrieved highest-scoring pairs were subject to phylogenetic reconstruction with fasttree 2.1.11 [47] and pairwise distance matrix calculation using the proportion of different sites between samples (‘raw’ distance) as implemented in the R package pegas [48].

To demonstrate the pipeline’s capability of genotyping large viral genomes, we re-analyzed four samples of Lassalle et al. (2020) [49] originally reconstructed using snippy [50]. Since our pipeline needed a high read depth for HSV-1 to perform better, we included four samples of Lassalle et al. (2020) [49] with a coverage larger than 3000×. For the genotyping we used the reference genome of Herpes simplex virus type 1 (NC_001806.2). The resulting consensus genomes were submitted to Genome Detectives Virus Tool 2.40 [42] (https://www.genomedetective.com/app/typingtool/virus/). Then, the consensus sequences output by QVG were compared with the consensus genomes obtained by Lassalle et al. (2020) [49] Whole-genome alignments were conducted using MAFFT 7.490 [51], and the pairwise distance matrix was calculated as shown for the Aviadenovirus and FCoV datasets.

## Results and discussion

**Fig 2:**
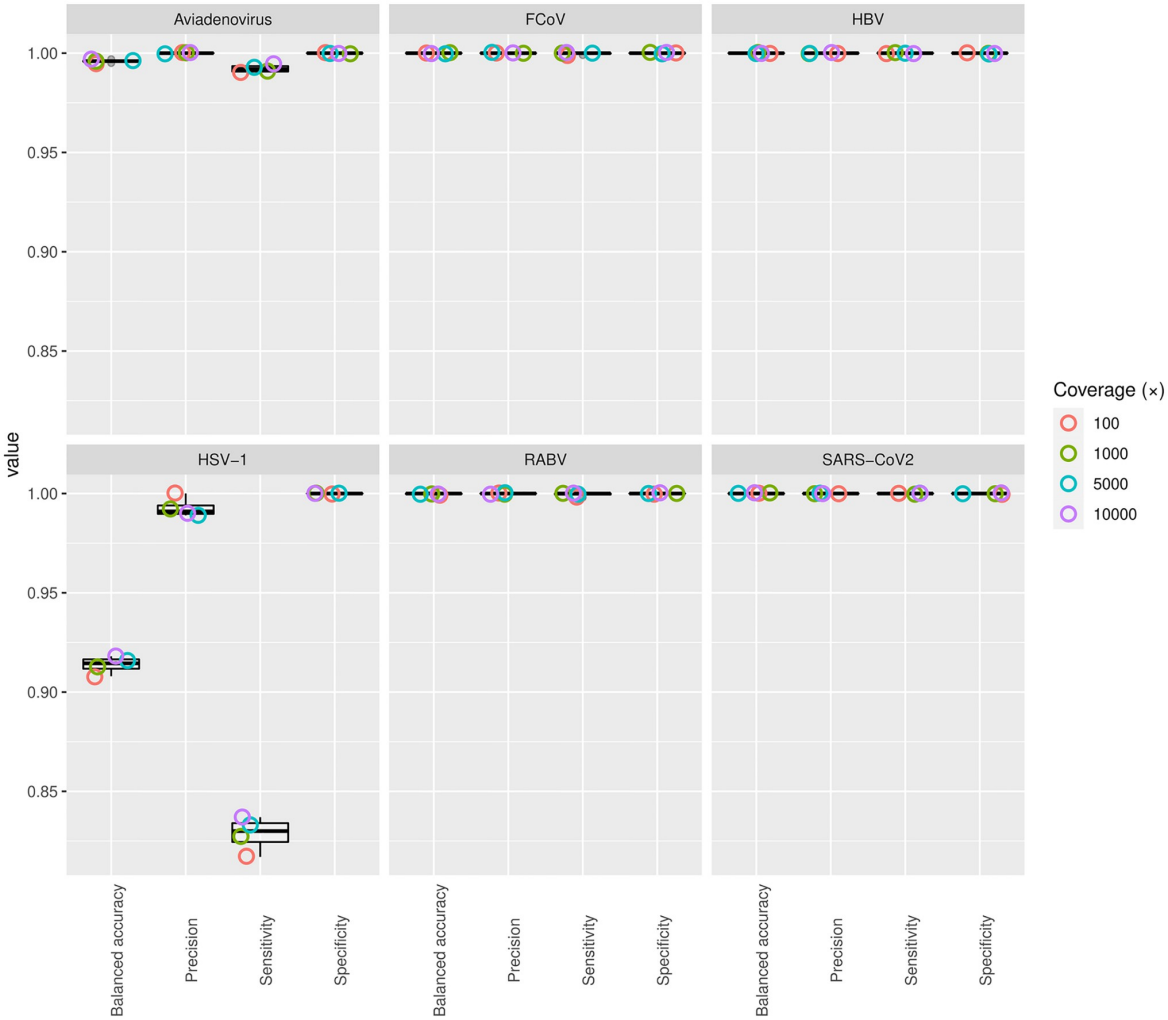
Statistical assessment of the presented pipeline’s accuracy. The plots show the values of sensitivity (true positive rate—TPR), specificity (true negative rate—TNR), balanced accuracy (BA), and precision (positive predictive value—PPV).

The analysis of the simulated datasets generally showed a high accuracy across datasets with a different coverage values (Fig 2). For the SARS-CoV2, HBV, RABV, and FCoV datasets regardless of coverage, QVG showed a sensitivity larger than 0.998 and a specificity, balanced accuracy and precision of 1.0. The adenovirus dataset showed an inflated number of false negatives, thus, decreasing sensitivity and balanced accuracy, both of which remained larger than 0.99 regardless of sequencing depth. All the false negative polymorphisms could be found in the ORF8 region of the reference genome. Inspecting the short-read alignments revealed ambiguous alignments with low mapping qualities (i.e. reads could be mapped to more than one different genomic region with an equal probability), which we linked to the false negative observations of mutations. The lowest sensitivity and balanced accuracy could be observed for the HSV-1 dataset (Fig 2). Although the lower sensitivity (0.82) could be somewhat mitigated by higher read depth, the sensitivity never exceeded 0.837. The specificity appeared to be 1.0 in every case, and we observed a precision higher than 0.98. This finding corroborates that repetitive genome content poses a challenge for the reference-based genotyping methods and might inflate the frequency of polymorphisms undiscovered due to the uncertainty of short-read alignments, a shortcoming of practically all widely used short-read aligner tools [12].

**Fig 3:**
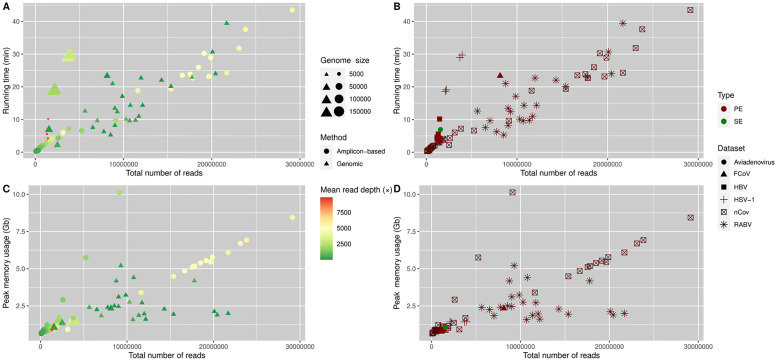
(A,B) Time (C,D) and memory required to run the whole pipeline. Time is reported in minutes (min), and peak memory usage is reported in Gigabytes (Gb). Running time corresponds to the wall clock time, and peak memory usage refers to the maximum resident size as reported by the ’time’ utility. This analysis was run on a commercial laptop with an Intel i7-4910MQ processor. Using more threads decreased the running time proportionally. On the left plots (A,C) the size of symbols is proportional to the reference genome size. Different symbols indicate the approach used for genome sequencing. The "genomic" approach includes whole genome, metagenomic and transcriptome sequencing. The symbol’s color represents mean read depth- The x-axis shows the number of reads supplied to the pipeline, including those that could not be aligned to the reference genome. On the right panels (B,D) the symbol’s color shows the type of the sequencing run, and different symbols indicate the sample’s corresponding dataset (Table 1). Runs shown on these plots used the annotation transfer feature of our pipeline alignment with resampling of alignments turned off.

We could obtain a good quality reference in all runs presented here. The most important factor to influence the total running time (including the quality filtering, read alignment, and variant calling) appeared to be the number of reads supplied to the pipeline, regardless of the sequencing approach (Fig 3A and 3B). The mean read depth of the samples affected the running time to a much lesser extent than the total number of reads (including those that did not align to the reference genome). The running time varied considerably; the SARS-CoV2 sample S5 generated for this study could be genotyped under 18 seconds, whereas the analysis of the SARS-CoV2 sample SRR14824569 needed the most time to finish, more than 43 minutes. Both extremities of running time used an amplicon-based approach to obtain sequencing reads. The genotyping of the samples relying on a genomic approach could be run in a similar time span. The only SARS-CoV2 sample relying on the transcriptomic approach (SRR17309642) was analyzed under two minutes, whereas the RABV sample SRR12012239 could be processed in 39 minutes (Fig 3A and 3B). The total running time could be decreased proportionally by using more CPU cores.

A similar relationship could be observed for the peak memory usage (Fig 3C and 3D). The main factor influencing memory usage appeared to be the number of reads supplied, and the mean read depth had a much smaller effect on memory usage. The minimum (0.63 Gb) and maximum (8.43 Gb) memory usage could be linked to the same samples as for the extremities of running time required to genotype the samples (S5 and SRR14824569). Since the variant calling step uses one thread for each sample, incrementing the number of CPU threads increased the memory usage only at this step, and the memory required for genotyping appeared to be additive (i.e. if more samples were genotyped simultaneously, all the memory needed to genotype those samples were allocated at the same time).

**Fig 4:**
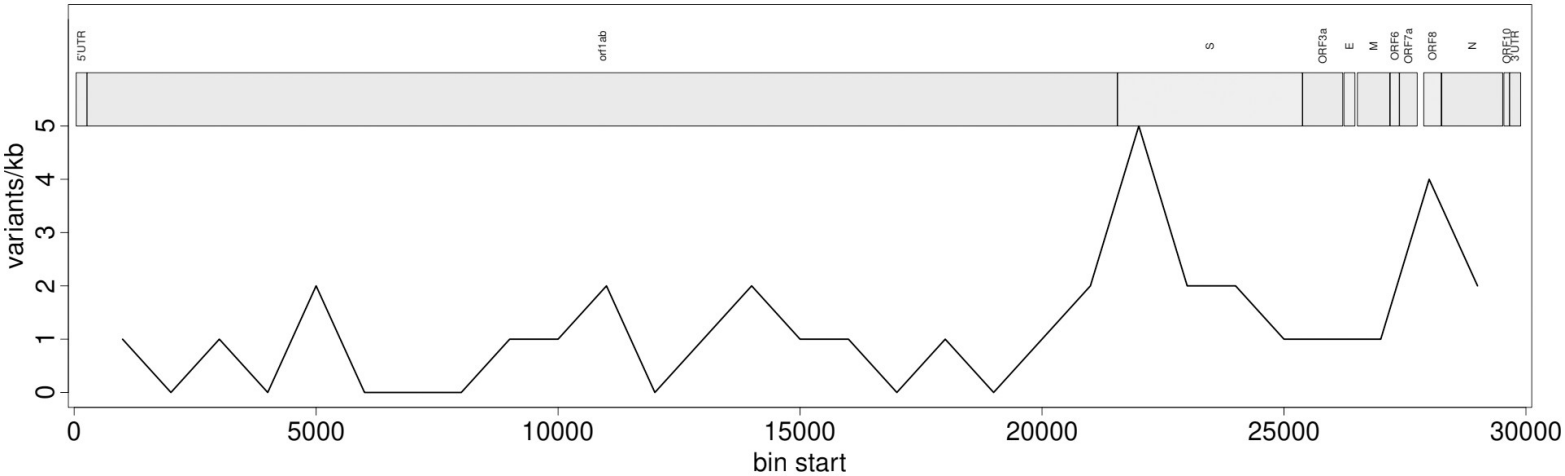
Example of the SNP density of sample S11 across the reference genome. The x-axis shows the genomic position, whereas the y-axis represents the number of SNPs within sliding windows.

**Fig 5:**
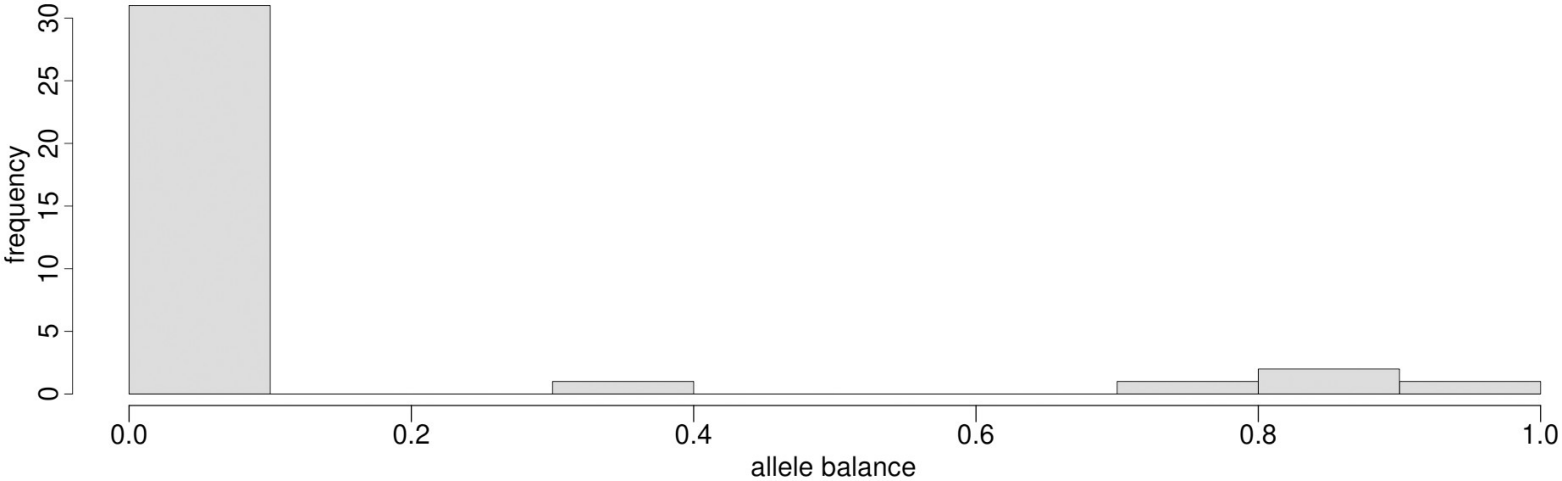
Example of AB distribution (sample S11) visualized as a histogram. An AB value different from 0 suggests multiple probable alleles at a given site.

Sequencing reads of the SARS-CoV2 dataset generated for this study covered 94.7–99.9% of the reference genome (S1 Table). The SNP density of all 20 samples appeared to be roughly equal across the genome, except at ORF8, in line with the findings of Flower et al. (2021) [52], and in the gene encoding the spike protein (S) that is known to harbor several mutations in the lineage AY.4 (Fig 4). Polymorphism within samples indicating more than one probable allele (Fig 5) could be found in all samples, but the same polymorphic site rarely showed an AB > 0 in more than one sample. Generally, 1–5 sites showed within-host variability. The only exceptions were two transitions at positions 21,987 and 24,410 found in 17 and 12 isolates, respectively. These are known but not characteristic mutations of the lineage identified by Pangolin. Submitting the alternative alleles to Pangolin did not change the result of the lineage assignment. The Pangolin lineage assignment using the consensus genome obtained by QVG and Geneious showed identical results and very similar support values, except for the sample S15. This sample using QVG could be assigned to the lineage AY.42, whereas using Geneious, it could be identified as AY.43. This discordance could be linked to this sample’s relatively lower sequencing breadth (S1 Table). Statistical support values were not unequivocally better for either pipeline (Table 2).

**Table 2:** Comparison of pipelines used in this study by the lineage assignment and support values as output by Pangolin. The only sample assigned differently after genotyping by the two compared pipelines is given in bold.

Sequence name	QVG						Geneious					
	Lineage	Conflict	Ambiguity score	Scorpio call	Scorpio support	Scorpio conflict	Lineage	Conflict	Ambiguity score	Scorpio call	Scorpio support	Scorpio conflict
S2	AY.4	0	1	Delta (AY.4-like)	0.91	0.06	AY.4	0	1.00	Delta (AY.4-like)	0.91	0.03
S3	AY.46.6	0	0.96	Delta (B.1.617.2-like)	0.85	0.15	AY.46.6	0	0.97	Delta (B.1.617.2-like)	0.92	0.08
S4	AY.46	0	0.99	Delta (B.1.617.2-like)	0.92	0.08	AY.39	0	0.99	Delta (B.1.617.2-like)	0.85	0.08
S5	AY.43	0	0.99	Delta (B.1.617.2-like)	0.92	0.08	AY.43	0	0.99	Delta (B.1.617.2-like)	0.92	0.08
S8	AY.4	0	1	Delta (AY.4-like)	0.91	0.06	AY.4	0	1	Delta (AY.4-like)	0.94	0.03
S9	AY.43	0	1	Delta (B.1.617.2-like)	0.92	0.08	AY.43	0	1	Delta (B.1.617.2-like)	0.92	0.08
S10	AY.43	0	1	Delta (B.1.617.2-like)	0.92	0.08	AY.43	0	1	Delta (B.1.617.2-like)	0.92	0.08
S11	AY.9.2	0	1	Delta (B.1.617.2-like)	0.92	0.08	AY.9.2	0	1	Delta (B.1.617.2-like)	1	0
S12	AY.9.1	0	1	Delta (B.1.617.2-like)	0.92	0.08	AY.9.1	0	1	Delta (B.1.617.2-like)	1	0
S13	AY.43	0	1	Delta (B.1.617.2-like)	0.92	0.08	AY.43	0	1	Delta (B.1.617.2-like)	0.92	0.08
S14	AY.43	0	1	Delta (B.1.617.2-like)	0.92	0.08	AY.43	0	1	Delta (B.1.617.2-like)	0.85	0.15
**S15**	**AY.42**	**0**	**0.94**	**Delta (B.1.617.2-like)**	**0.69**	**0.15**	**AY.43**	**0**	**0.96**	**Delta (B.1.617.2-like)**	**0.85**	**0**
S16	AY.3	0	1	Delta (B.1.617.2-like)	0.92	0.08	AY.3	0	1	Delta (B.1.617.2-like)	0.92	0.08
S17	AY.43	0	1	Delta (B.1.617.2-like)	0.92	0.08	AY.43	0	1	Delta (B.1.617.2-like)	0.92	0.08
S18	AY.122	0	1	Delta (B.1.617.2-like)	0.92	0.08	AY.122	0	1	Delta (B.1.617.2-like)	0.92	0.08
S19	AY.46.6	0	1	Delta (B.1.617.2-like)	1	0	AY.46.6	0	1	Delta (B.1.617.2-like)	1	0
S20	AY.43	0	1	Delta (B.1.617.2-like)	0.92	0.08	AY.43	0	1	Delta (B.1.617.2-like)	0.92	0.08
S22	AY.43	0	1	Delta (B.1.617.2-like)	1	0	AY.43	0	1	Delta (B.1.617.2-like)	1	0
S23	AY.122	0	0.99	Delta (B.1.617.2-like)	0.92	0.08	AY.122	0	0.99	Delta (B.1.617.2-like)	0.92	0.08
S24	AY.122	0	1	Delta (B.1.617.2-like)	1	0	AY.122	0	1	Delta (B.1.617.2-like)	1	0

**Fig 6:**
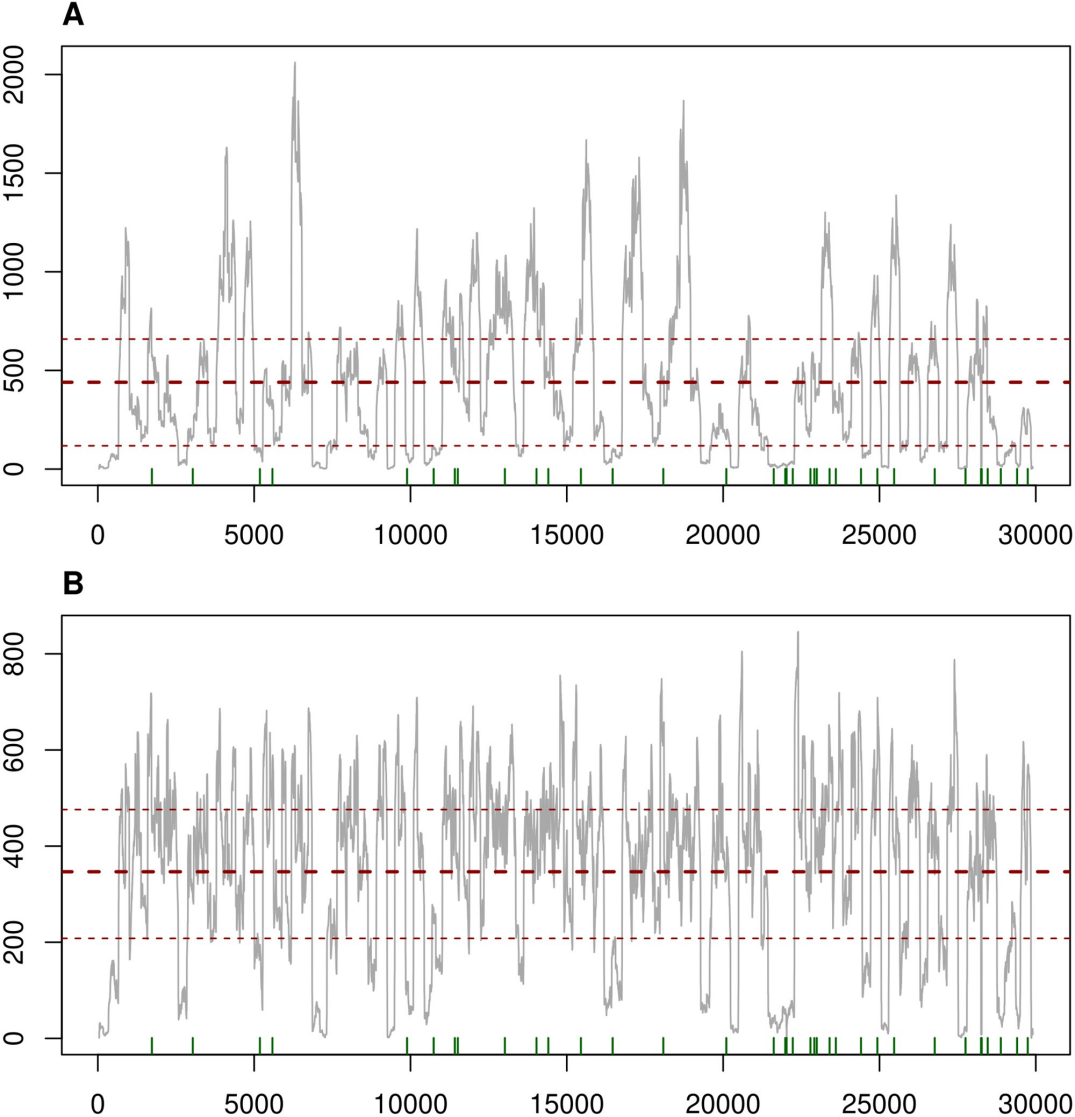
(A) Example of read depth counting all alignments and (B) evened out read depth by the resampling feature of using our pipeline. The x-axis shows the genomic position, whereas the y-axis represents the read depth of each position, shown as a gray line. The middle red dashed line shows the mean of read depth across the genome, and the thinner dashed lines show the first and third quartile of read depth distribution. Green bars on the x-axis show the positions of polymorphisms discovered using all alignments (A) and the read depth after resampling the alignments along genomic windows (B).

We observed a much greater unevenness of read depth in the SARS-CoV-2 sequencing reads than in any other dataset. The resampling of alignments in 100 bp windows efficiently evened out the read depth along the reference genome. Using S11 of our SARS-CoV-2 as an example, with this feature turned on, we could decrease the range of read depth from 1–2061 (mean = 346.742) to 1–846 (mean = 440.111), not counting sites with a depth of zero, which eliminated the "spikes" of high read depth regions (Fig 6). The resampling of alignments tended to increase total running time by up to 70% (mean = 28.6%), which change of running time was not related to the mean read depth. This option had a much more pronounced effect on the memory usage of the pipeline. The resampling to an even read depth reduced the memory usage of genotyping of samples with a mean read depth > 4000× by up to 371.84% (mean = 226.98%). Although the smoothing of read depth did not affect the number and identity of discovered polymorphisms for none of the SARS-CoV-2 samples, together with the clipping of high-depth alignment positions, this feature can potentially aid in eliminating false positive polymorphisms found due to read-depth biases and decrease the memory usage at the same time.

The publicly available SARS-CoV2 sequencing data showed similar results. The breadth varied between 97.1–100% (S2 Table). Similar to the dataset generated for this study, SNPs showed the highest density at the spike protein and ORF8. Only two samples did not show signs of within-host diversity (SRR16912539, SRR16912480). Other samples showed 1–15 polymorphisms with an AB > 0, of which SNPs at positions 28,270 could be found in 11 samples, whereas such polymorphisms at positions 28,095 and 29,870 were found in 4–4 samples. The identities of the consensus genomes always matched with the already published identification (Table 3). Only sample SRR14824567 was classified as a different lineage (B.1.637) than the original lineage (B.1.526.1)., but later this B.1.526.1 was designated to B.1.637 in Pangolin. Despite these samples having a various number of reads, mean read depth, and being sequenced using different approaches (Table 1 and S2 Table), our pipeline outputs good quality consensus genomes.

**Table 3:** Comparison of originally reported lineages and lineages identified by Pangolin after genotyping publicly available sequencing reads of SARS-CoV2 with our pipeline.

Sequence name	Lineage	Conflict	Ambiguity score	Scorpio call	Scorpio support	Scorpio conflict	Originally reported lineage
SRR14155371	B.1.1.7	0	0.98	Alpha (B.1.1.7-like)	0.96	0.04	B.1.1.7
SRR14155385	B.1.1.7	0	1.0	Alpha (B.1.1.7-like)	0.96	0.04	B.1.1.7
SRR14824560	B.1.1.7	0	0.98	Alpha (B.1.1.7-like)	0.96	0.04	B.1.1.7
SRR14824561	B.1.1.7	0	0.98	Alpha (B.1.1.7-like)	0.96	0.04	B.1.1.7
SRR14824562	B.1.429	0	1.0	Epsilon (B.1.429-like)	1.0	0	B.1.429
SRR14824563	P.1	0	1.0	Gamma (P.1-like)	0.87	0	P.1
SRR14824564	B.1.1.7	0	1.0	Alpha (B.1.1.7-like)	0.91	0.04	B.1.1.7
SRR14824565	B.1.1.7	0	1.0	Alpha (B.1.1.7-like)	0.96	0.04	B.1.1.7
SRR14824566	P.1	0	1.0	Gamma (P.1-like)	0.87	0	P.1
SRR14824567	B.1.637	0	1.0				B.1.526.1
SRR14824568	B.1.1.7	0	1.0	Alpha (B.1.1.7-like)	0.95	0.04	B.1.1.7
SRR14824569	B.1.1.7	0	1.0	Alpha (B.1.1.7-like)	0.95	0.04	B.1.1.7
SRR14824570	B.1.1.7	0	1.0	Alpha (B.1.1.7-like)	0.95	0.04	B.1.1.7
SRR14824572	B.1.525	0	0.98	Eta (B.1.525-like)	1.00	0	B.1.525
SRR14824573	B.1.1.7	0	1.0	Alpha (B.1.1.7-like)	0.96	0.04	B.1.1.7
SRR14824574	B.1.1.7	0	1.0	Alpha (B.1.1.7-like)	0.96	0.04	B.1.1.7
SRR16741159	B.1.351	0	0.98	Beta (B.1.351-like)	0.78	0.14	B.1.351
SRR16912480	P.1	0	1.0	Gamma (P.1-like)	0.87	0	P.1
SRR16912539	P.1	0	1.0	Gamma (P.1-like)	0.87	0	P.1
SRR17309642	BA.1	0	1.0	Omicron (BA.1-like)	0.91	0	B.1.1.529/Omicron

**Fig 7:**
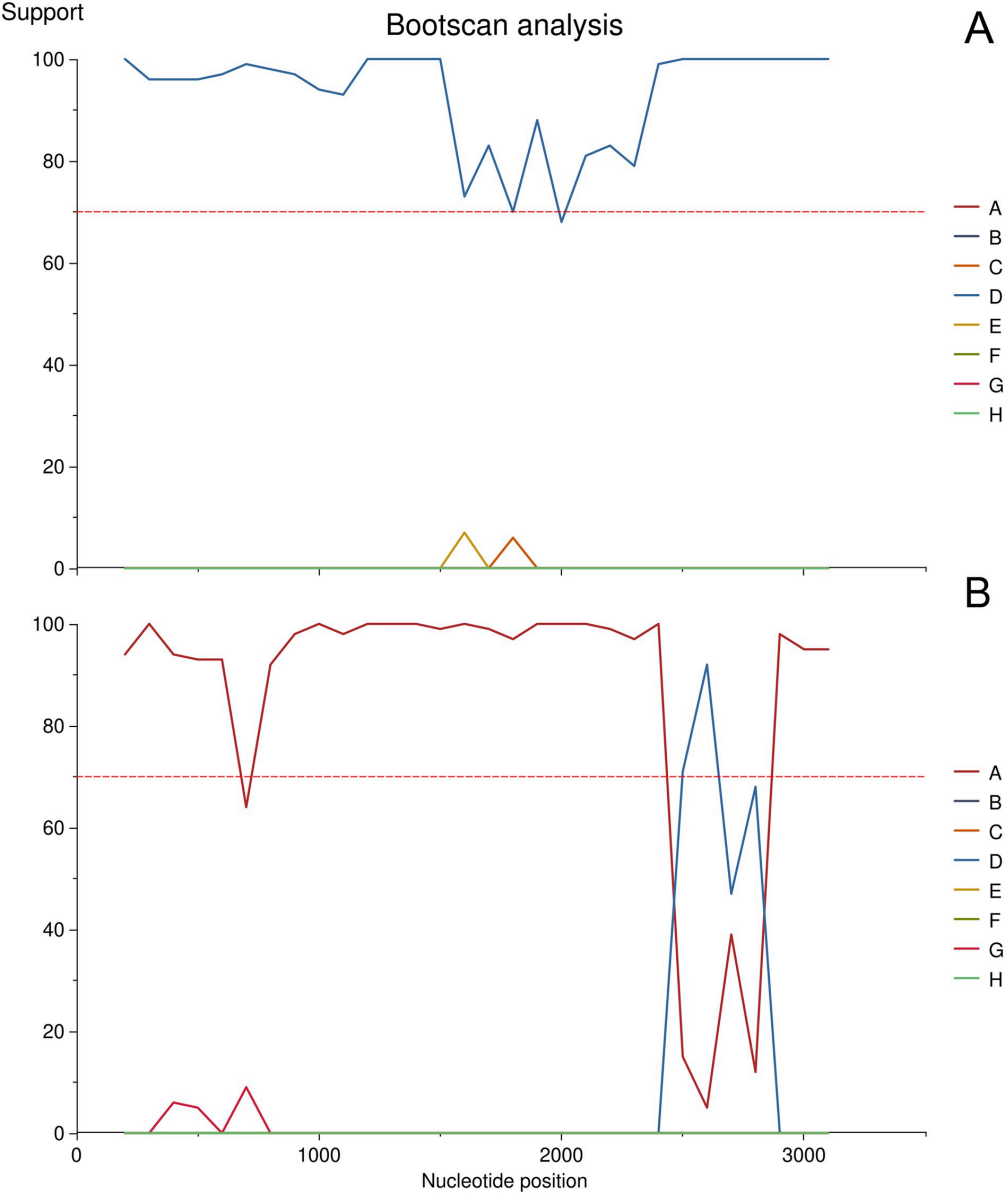
(A) Example of an unequivocally identified HBV sample (SRR12535946) and (B) a recombinant sample (SRR12535947) as output by Genome Detective using Bootscan. Values on the y-axis show positions of x belonging to a given cluster.

The sequencing breadth of the HBV dataset showed a higher variability (65.7–100%). SNP density in 1,000 bp windows peaked at 84 (sample SRR12535947) and generally showed a decreasing trend towards the end position of the reference genome. Samples had 1–28 polymorphic sites with more than one probable allele. The same ’non-haploid’ (i.e. multiple probable alleles could be observed) position could be observed in a maximum of two samples. Genome Detective could correctly assign genomes into HBV subtypes, except for one sample (Table 4). The bootscan analysis (Fig 7) confirmed that the dominant genome, which could not be equivocally assigned to any lineage, can be a recombinant of strains A and D. Recombination is not unprecedented for HBV [53–55] and can play an important role in the evolution of HBV genotypes [53].

**Table 4:** Short result of the phylogenetic type classification of HBV samples by genome detective.

Name	Length	Begin	End	Species	Type	Type support	Original subtype
SRR12535936	3182	1	3182	Hepatitis B virus	subtype D	100.0	subtype D
SRR12535937	3179	4	3182	Hepatitis B virus	subtype D	100.0	subtype D
SRR12535938	3182	1	3182	Hepatitis B virus	subtype D	100.0	subtype D
SRR12535946	3182	1	3182	Hepatitis B virus	subtype D	100.0	subtype D
SRR12535947	3179	1	3182	Hepatitis B virus	Could not assign		subtype A

The breadth of RABV samples appeared to be at least 98.76%. Since the genomic approach applied to obtain the sequencing reads of this dataset does not strictly rely on species-specific PCR amplicons, the mean read depth (S4 Table) was lower than for previously described datasets. SNP density of the dataset varied between 11–37 and showed a roughly uniform distribution within samples. Seven out of 23 samples showed no variants with an AB > 0 (SRR12012247, SRR12012251, SRR12012242, SRR12012245, SRR12012250, SRR12012240, SRR12012237, SRR12012254). The remaining samples had 1–14 ‘non-haploid’ sites, and the same such site could be observed in a maximum of two samples. RABV-GLUE identified the samples as the cosmopolitan AF1b lineage (Table 5), agreeing with the originally reported classification [44].

**Table 5:** Result of classification of the RABV datasets samples returned by RABV-GLUE.

					Coding region coverage					
Sequence	Identified as RABV?	Major clade	Minor clade	Closest full genome reference sequence	N (%)	P (%)	M (%)	G (%)	L (%)	Originally reported lineage
SRR12012234	Yes	Cosmopolitan	Cosmopolitan AF1b	KX148204	100	100	100	100	100	Africa 1-b lineage
SRR12012235	Yes	Cosmopolitan	Cosmopolitan AF1b	KX148103	100	100	100	100	100	Africa 1-b lineage
SRR12012236	Yes	Cosmopolitan	Cosmopolitan AF1b	KX148204	100	100	100	100	100	Africa 1-b lineage
SRR12012237	Yes	Cosmopolitan	Cosmopolitan AF1b	KX148103	100	100	100	100	100	Africa 1-b lineage
SRR12012238	Yes	Cosmopolitan	Cosmopolitan AF1b	KX148103	100	100	100	100	100	Africa 1-b lineage
SRR12012239	Yes	Cosmopolitan	Cosmopolitan AF1b	KX148103	100	100	100	100	100	Africa 1-b lineage
SRR12012240	Yes	Cosmopolitan	Cosmopolitan AF1b	KX148103	100	100	100	100	100	Africa 1-b lineage
SRR12012241	Yes	Cosmopolitan	Cosmopolitan AF1b	KX148103	100	100	100	100	100	Africa 1-b lineage
SRR12012242	Yes	Cosmopolitan	Cosmopolitan AF1b	KX148103	100	100	100	100	100	Africa 1-b lineage
SRR12012243	Yes	Cosmopolitan	Cosmopolitan AF1b	KX148103	100	100	100	100	100	Africa 1-b lineage
SRR12012244	Yes	Cosmopolitan	Cosmopolitan AF1b	KX148204	100	100	100	100	100	Africa 1-b lineage
SRR12012245	Yes	Cosmopolitan	Cosmopolitan AF1b	KX148204	100	100	100	100	100	Africa 1-b lineage
SRR12012246	Yes	Cosmopolitan	Cosmopolitan AF1b	KX148204	100	100	100	100	100	Africa 1-b lineage
SRR12012247	Yes	Cosmopolitan	Cosmopolitan AF1b	KX148204	100	100	100	100	100	Africa 1-b lineage
SRR12012248	Yes	Cosmopolitan	Cosmopolitan AF1b	KX148103	100	100	100	100	100	Africa 1-b lineage
SRR12012249	Yes	Cosmopolitan	Cosmopolitan AF1b	KX148204	100	100	100	100	100	Africa 1-b lineage
SRR12012250	Yes	Cosmopolitan	Cosmopolitan AF1b	KX148204	100	100	100	100	100	Africa 1-b lineage
SRR12012251	Yes	Cosmopolitan	Cosmopolitan AF1b	KX148204	100	100	100	100	100	Africa 1-b lineage
SRR12012252	Yes	Cosmopolitan	Cosmopolitan AF1b	KX148204	100	100	100	100	100	Africa 1-b lineage
SRR12012253	Yes	Cosmopolitan	Cosmopolitan AF1b	KX148204	100	100	100	100	100	Africa 1-b lineage
SRR12012254	Yes	Cosmopolitan	Cosmopolitan AF1b	KX148103	100	100	100	100	100	Africa 1-b lineage
SRR12012255	Yes	Cosmopolitan	Cosmopolitan AF1b	KX148204	100	100	100	100	100	Africa 1-b lineage
SRR12012256	Yes	Cosmopolitan	Cosmopolitan AF1b	KX148103	100	100	100	100	100	Africa 1-b lineage

**Fig 8:**
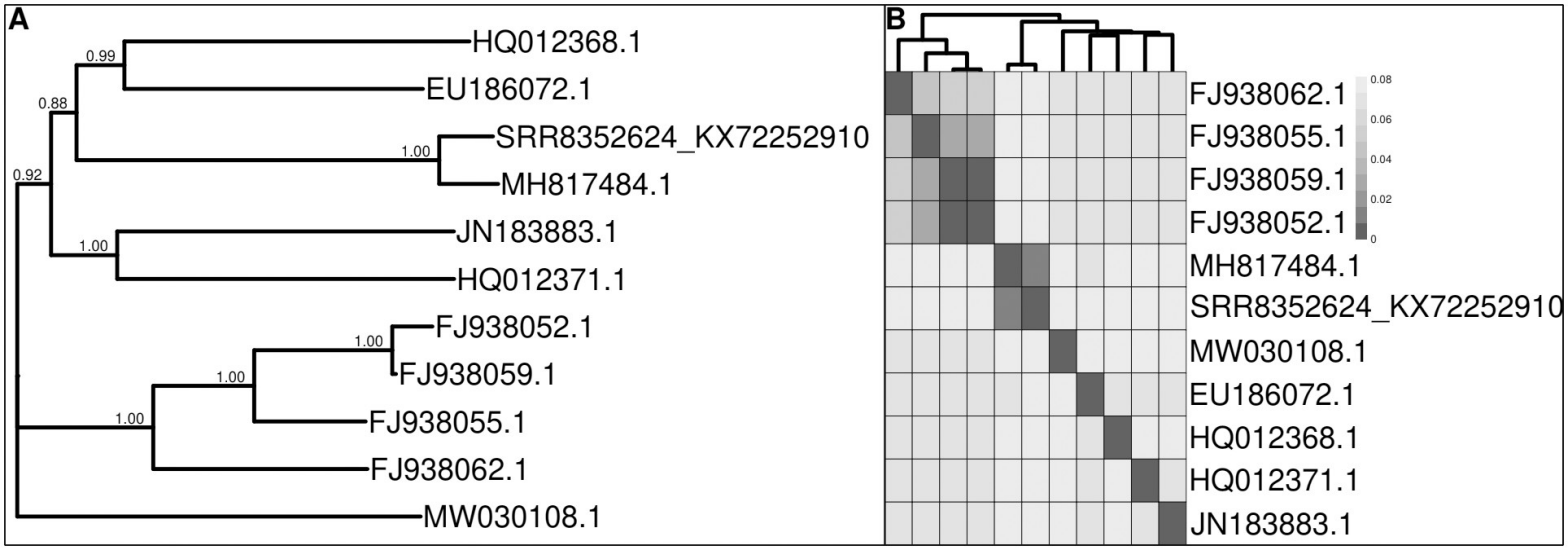
(A) Phylogenetic tree reconstructed for the best 10 BLAST hits using the consensus FCoV genome obtained using our pipeline and (B) pairwise sequence similarity shown on a heatmap of these sequences using raw distances (B). The sample name SRR8352624_KX72252910 represents the sequencing reads genotyped with our pipeline relying on alignments to the reference genome KX722529.1 and MH817484 shows the position of the publicly available reference genome of feline coronavirus strain FCoV-SB22[45].

Sequencing reads of the FCoV sample covered more than 94% of the reference genome but showed the lowest read depth of all samples analyzed in this study (S5 Table). The SNP density peaked at 73 and showed multiple highly polymorphic islands along the reference genome. In total, 72 out of 1,411 polymorphic sites showed within-host diversity based on AB values. The phylogenetic reconstruction correctly placed the consensus genome output by QVG closest to the publicly available genome (Fig 8A) of the same sample. However, a relatively higher genetic distance could be observed between these two sequences (Fig 8A and 8B). We link this phenomenon to the relatively low read depth of the sequencing reads (mean = 4.87), which can decrease reference-based genotyping accuracy. The low read depth can be a limitation of the approach presented here and any reference-based genotyping method.

**Fig 9:**
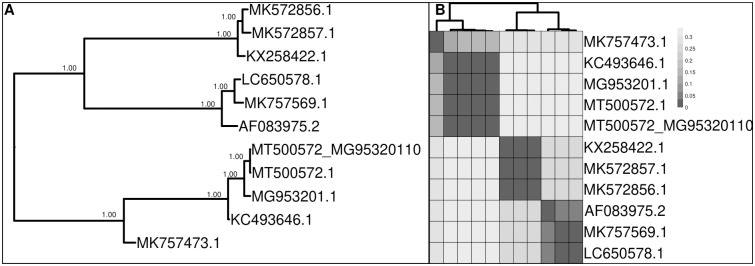
(A) Phylogenetic tree reconstructed for the best 10 BLAST hits using the consensus avian Adenovirus genome obtained using our pipeline and (B) pairwise sequence similarity shown on a heatmap of these sequences using raw distances. The sample name MT500572_MG95320110 represents the sequencing reads genotyped with our pipeline relying on alignments to the reference genome MG953201.1, and MT500572.1 shows the position of the publicly available reference genome of the avian adenovirus isolate D2453/1/10-12/13/UA [46].

The obtained consensus genome of the adenovirus sample covered 99.4% of the reference genome (S6 Table). The SNP density appeared to be higher in the pVI, ORF22, and ORF17—ORF19A genes relative to the rest of the genome. In total, we observed seven sites with an AB > 0. The phylogenetic reconstruction clustered the consensus genome genotyped here and the publicly available genome of the same sample (Fig 9A), agreeing with the clustering based on pairwise genetic distances (Fig 9B). We only observed indel mutations between the two mentioned sequences that could be linked to the automatic masking of low-depth genomic regions (read depth < 5). This low divergence of the reference points out the accuracy of the presented pipeline.

**Fig 10:**
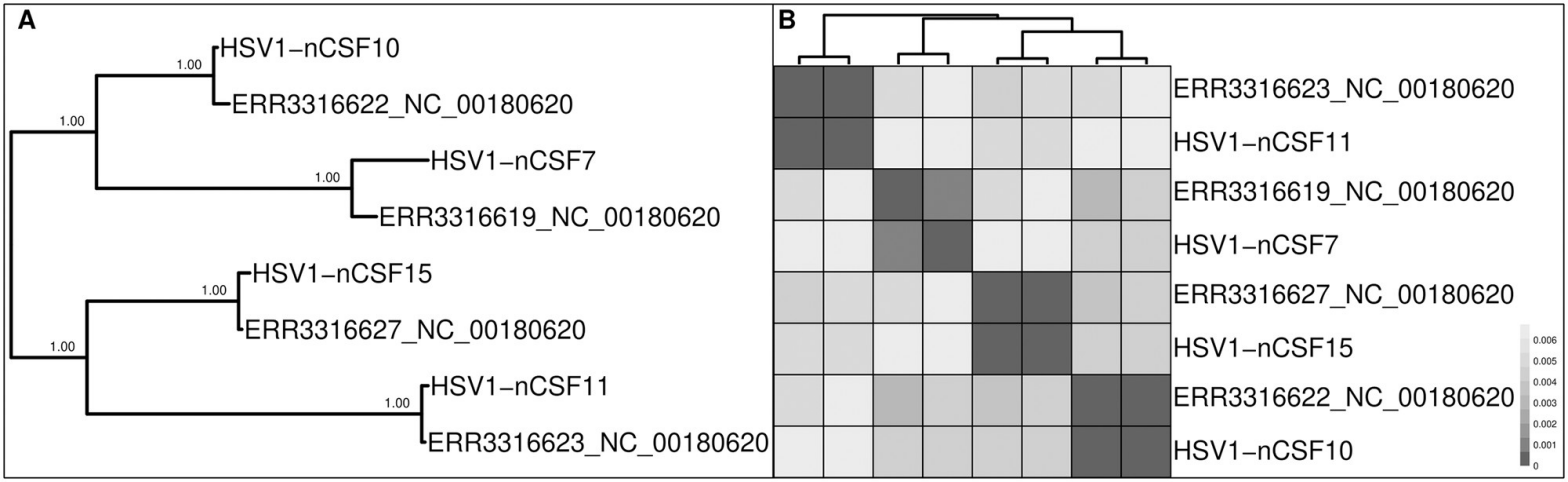
(A) Phylogenetic tree reconstructed for the HSV-1 dataset and (B) heatmap showing the pairwise distances of genome consensus sequences. Sample names starting with "HSV-1" represent the sequences reconstructed by Lassalle et al. (2020) [49], and accession numbers show the placement of the newly reconstructed genome sequences of the same samples. The accession of the reference genome used for the analysis is given next to the accession number of raw read data.

The HSV-1 sequencing reads covered more than 98.16% of the reference genome (S7 Table). The SNP density appeared to be even without any obvious peaks, except for the genes gG (US4) and gI (US7), which are among the most diverse genes of alphaherpesviruses [56]. We observed two "non-haploid" sites occurring in all four samples. One such site could be observed in three, and six of them occurred in two samples. The majority of sites with AB > 0 (n = 179) were unique to one sample. Genome detective correctly identified the consensus genomes as HSV-1 sequences with a concordance of > 99.12%. This tool identified 77 CDS sequences with 77 stop codons for three samples. The UL24 gene of ERR3316619 showed an extra stop codon due to a T>G mutation also present in the published sequence (HSV1-nCSF7) of Lassalle et al. (2020) [49]. The phylogenetic reconstruction (Fig 10A), in agreement with the pairwise distance-based clustering (Fig 10B), correctly placed the newly generated consensus genome sequences at a low genetic distance from the corresponding public accession of the same sample. These results suggest a high accuracy of the consensus genome sequences, despite the lower accuracy detected for large and repetitive genomes (such as HSV-1) using the synthetic dataset (Fig 2).

This work reports a pipeline capable of rapid and automated analysis of viral genomes obtained by NGS. Unlike proprietary software solutions, this pipeline relies on freely available, open-source bioinformatic software. Using parallel execution of tasks, we could obtain consensus genomes of the SARS-CoV2 dataset generated for this study without the need for laborious manual data curation required by Geneious and with similar accuracy. Our pipeline generated good quality consensus genomes using its default settings in most cases, with the FCoV sample as the only exception. Moreover, we could also investigate the intra-host diversity of samples using the allele balance values. The occurrence of the same variable sites sharing more probable identical alternative alleles within datasets showed that these ‘non-haploid’ polymorphisms are probably existing mutations originating from multiple acquisitions of different strains.

We genotyped already known genomes of six viral species. Some of these viruses are highly variable (HBV, HSV-1, SARS-CoV-2) and can pose dangers to humans and domestic or wild animals (Aviadenoviurs, RABV, FcoV); thus, it can be important to identify them and track their molecular evolution. All samples genotyped by our pipeline were correctly identified by classification tools, except one HBV sample, which appeared to be a recombinant genome. Our results demonstrate that QVG can handle a wide range of Illumina sequencing platforms (NextSeq, MiniSeq, MiSeq, HiSeq 2500, NovaSeq 6000), different genome sizes (3182–152,252 bp), a broad range of short read lengths (76–250 bp). However, care should be taken to set the correct parameters if the sequencing breadth or the mean read depth is relatively lower. The only inconsistency between genotyping approaches (S15 of the SARS-CoV2 dataset) and an inflated genetic distance (FCoV) could be linked to these issues.

Currently, the pipeline presented does not include any specific step to remove contamination and assumes that the target viral DNA is present in the highest frequency and the low-frequency polymorphisms originating from contaminants and/or sequencing error are removed during variant call. Since the enrichment of viruses [57] is frequently applied prior to sequencing or targeted sequencing is carried on, we believe the possible low-frequency contaminants would not distort the results of QVG. Our pipeline’s obvious shortcoming is that samples’ characterization relies on a closely related reference genome, which, if not yet available, should be assembled first using, e.g. VirusTAP [58] or V-ASAP [2]. QVG expects one specific reference genome for the analysis. The genotyping of multiple enriched samples might need the repeated run of QVG, as this tool is designed for the genotyping of one targeted virus genome. Only the reads aligned to the reference will be used for the analysis, and the rest of the sequencing reads will not be kept in the dataset.

Nevertheless, we showed that QVG is able to analyze viral genome sequencing datasets in a short time without any user intervention, promoting the quick analysis of samples, which might be an important aspect of high throughput data generation and processing. With the design presented in this study, we were able to obtain high-accuracy consensus genomes suitable for downstream analyses. The presented pipeline utilizes the quality filtering of reads, the filtering of polymorphisms by their read depth ratio, and the quality of called polymorphisms to achieve its performance. Moreover, by annotation transfer, the newly obtained consensus genomes could be automatically annotated without manual curation. The analysis of allele balance after genotype calling with ploidy unset makes the analysis of within-host variation feasible. The fine-tuning capability via the wide range of command-line options allows the adaptation of QVG to a wide range of datasets, including amplicon-based and (meta)genomic sequencing data. Although the usage of reads shorter than 72 bp with the default short-read alignment parameters can increase the ratio of ambiguous alignments, the issue might be mitigated by setting the alignment parameters (minimum seed length, matching score, mismatch, gap open, gap extension, and clipping penalties) from the command line. The setting of appropriate alignment parameters can be of particular importance for the analysis of ancient viral DNA due to, among other things, *post-mortem* DNA degradation and contamination [59], resulting in potentially shorter read lengths. Despite these difficulties, the number of discovered ancient viruses is constantly increasing (e.g. [60]). With the convenient setting of alignment parameters, the flexibility of our pipeline can potentially allow the reconstruction of ancient viral sequences.

Our pipeline can be installed conveniently on any computer running a UNIX-like operating system, for which instructions and detailed documentation are given on the project GitHub page (https://github.com/laczkol/QVG). The free availability at GitHub also ensures transparency and modifiability of QVG and is a great option to receive community feedback about the usage and potential issues of the pipeline. Given the above, we believe that QVG can be a viable alternative to other, existing tools, such as TRACESPipe [7] and nfcore-viralrecon [8, 9] and V-pipe [14]. Matched with the speed of NGS techniques, QVG can be an important and valuable tool for the mass analysis of viral samples and for tracking outbreaks by identifying viral strains and checking the within-host diversity of samples. Brandt et al. (2021) [61] showed that the long-read sequencing technology (such as Oxford Nanopore) could be an efficient alternative to the Illumina platform for the reference-based genotyping of SARS-CoV2. Future directions of the pipelines development include the adaptation of long-read sequencing technology in our framework, targeted metagenomic processing of multiple genomes coupled with contamination control, and automatic lineage assignment.

## Supporting information

S1 Table Detailed statistics as exported with samtools coverage for the SARS-CoV2 dataset generated for this study. (DOCX)

S2 Table Detailed statistics as exported with samtools coverage for the publicly available SARS-CoV2 dataset. (DOCX)

S3 Table Detailed statistics as exported with samtools coverage for the HBV dataset. (DOCX)

S4 Table Detailed statistics as exported with samtools coverage for the RABV dataset. (DOCX)

S5 Table Detailed statistics as exported with samtools coverage for the FCoV sample. (DOCX)

S6 Table Detailed statistics as exported with samtools coverage for the avian adenovirus sample. (DOCX)

S7 Table Detailed statistics as exported with samtools coverage for the HSV-1 dataset. (DOCX)
