# Supplementary material for: Rapid genotyping of targeted viral samples using Illumina short-read sequencing data
Source: PLoS One. 2022 Sep 16;17(9):e0274414. doi: 10.1371/journal.pone.0274414 (PMC9481040; doi:10.1371/journal.pone.0274414)
Supplement: S1 Table — (DOCX) [file pone.0274414.s001.docx]

**S1 Table. Detailed statistics as exported with samtools coverage for the SARS-CoV2 dataset generated for this study.**

| sample_id | rname | startpos | endpos | numreads | covbases | coverage | meandepth | meanbaseq | meanmapq |
| --- | --- | --- | --- | --- | --- | --- | --- | --- | --- |
| S23 | MN908947.3 | 1 | 29903 | 194779 | 29652 | 99.16 | 666.72 | 37 | 60 |
| S11 | MN908947.3 | 1 | 29903 | 125861 | 29841 | 99.79 | 439.20 | 37 | 60 |
| S16 | MN908947.3 | 1 | 29903 | 370634 | 29847 | 99.81 | 1148.68 | 36.7 | 60 |
| S24 | MN908947.3 | 1 | 29903 | 491715 | 29869 | 99.88 | 1623.84 | 37 | 60 |
| S10 | MN908947.3 | 1 | 29903 | 822494 | 29865 | 99.87 | 2504.90 | 37 | 60 |
| S5 | MN908947.3 | 1 | 29903 | 55226 | 29449 | 98.48 | 173.48 | 36.9 | 59.9 |
| S12 | MN908947.3 | 1 | 29903 | 454599 | 29889 | 99.95 | 1534.75 | 37 | 60 |
| S9 | MN908947.3 | 1 | 29903 | 1021009 | 29900 | 99.99 | 3301.26 | 37.1 | 60 |
| S14 | MN908947.3 | 1 | 29903 | 1578737 | 29869 | 99.88 | 4621.05 | 37.1 | 60 |
| S15 | MN908947.3 | 1 | 29903 | 168510 | 28308 | 94.66 | 546.672 | 37 | 60 |
| S20 | MN908947.3 | 1 | 29903 | 871661 | 29891 | 99.96 | 2874.43 | 37.1 | 60 |
| S17 | MN908947.3 | 1 | 29903 | 924839 | 29873 | 99.90 | 2878.11 | 37.1 | 59.9 |
| S8 | MN908947.3 | 1 | 29903 | 380861 | 29896 | 99.98 | 1181.52 | 37 | 60 |
| S13 | MN908947.3 | 1 | 29903 | 560779 | 29865 | 99.88 | 1659.33 | 37 | 60 |
| S22 | MN908947.3 | 1 | 29903 | 330740 | 29869 | 99.89 | 1107.72 | 37.1 | 60 |
| S3 | MN908947.3 | 1 | 29903 | 21447 | 28692 | 95.95 | 72.82 | 36.9 | 58.8 |
| S4 | MN908947.3 | 1 | 29903 | 333836 | 29806 | 99.67 | 1094.15 | 36.9 | 60 |
| S2 | MN908947.3 | 1 | 29903 | 441063 | 29872 | 99.90 | 1381.93 | 37 | 60 |
| S18 | MN908947.3 | 1 | 29903 | 735956 | 29870 | 99.89 | 2424.97 | 36.8 | 60 |
| S19 | MN908947.3 | 1 | 29903 | 605008 | 29888 | 99.95 | 2178.96 | 37 | 60 |
