# Supplementary material for: Rapid genotyping of targeted viral samples using Illumina short-read sequencing data
Source: PLoS One. 2022 Sep 16;17(9):e0274414. doi: 10.1371/journal.pone.0274414 (PMC9481040; doi:10.1371/journal.pone.0274414)
Supplement: S2 Table — (DOCX) [file pone.0274414.s002.docx]

**S2 Table. Detailed statistics as exported with samtools coverage for the publicly available SARS-CoV2 dataset.**

| sample_id | rname | startpos | endpos | numreads | covbases | coverage | meandepth | meanbaseq | meanmapq |
| --- | --- | --- | --- | --- | --- | --- | --- | --- | --- |
| SRR14824562 | MN908947.3 | 1 | 29903 | 1770168 | 29903 | 100 | 4134.01 | 36.5 | 60 |
| SRR14824574 | MN908947.3 | 1 | 29903 | 1875745 | 29902 | 99.99 | 4351.20 | 36.6 | 60 |
| SRR14824564 | MN908947.3 | 1 | 29903 | 2453593 | 29896 | 99.97 | 5653.89 | 36.3 | 59.9 |
| SRR14824569 | MN908947.3 | 1 | 29903 | 2452509 | 29898 | 99.98 | 5641.75 | 36.6 | 60 |
| SRR14824568 | MN908947.3 | 1 | 29903 | 2153502 | 29865 | 99.87 | 4966.50 | 36.5 | 60 |
| SRR14824560 | MN908947.3 | 1 | 29903 | 626792 | 29659 | 99.18 | 1460.90 | 36.5 | 60 |
| SRR14824573 | MN908947.3 | 1 | 29903 | 2006313 | 29890 | 99.96 | 4634.16 | 36.6 | 60 |
| SRR14824572 | MN908947.3 | 1 | 29903 | 802442 | 29053 | 97.16 | 1861.43 | 36.6 | 59.9 |
| SRR14824570 | MN908947.3 | 1 | 29903 | 2098095 | 29895 | 99.97 | 4858.03 | 36.4 | 60 |
| SRR14824566 | MN908947.3 | 1 | 29903 | 2088873 | 29877 | 99.91 | 4820.52 | 36.5 | 60 |
| SRR14155385 | MN908947.3 | 1 | 29903 | 2050770 | 29901 | 99.99 | 4584.86 | 36.5 | 59.9 |
| SRR16912539 | MN908947.3 | 1 | 29903 | 114026 | 29903 | 100 | 431.565 | 36.3 | 60 |
| SRR14824563 | MN908947.3 | 1 | 29903 | 2363754 | 29889 | 99.95 | 5446.88 | 36.6 | 60 |
| SRR16741159 | MN908947.3 | 1 | 29903 | 964213 | 29039 | 97.11 | 2230.50 | 36.6 | 59.8 |
| SRR14824561 | MN908947.3 | 1 | 29903 | 2212633 | 29691 | 99.29 | 5113.92 | 36.5 | 60 |
| SRR16912480 | MN908947.3 | 1 | 29903 | 141449 | 29903 | 100 | 529.432 | 36.5 | 60 |
| SRR14824567 | MN908947.3 | 1 | 29903 | 874103 | 29892 | 99.96 | 2030.72 | 36.3 | 60 |
| SRR14824565 | MN908947.3 | 1 | 29903 | 2175466 | 29899 | 99.99 | 5027.86 | 36.6 | 60 |
| SRR17309642 | MN908947.3 | 1 | 29903 | 314900 | 29870 | 99.89 | 488.517 | 37.9 | 60 |
| SRR14155371 | MN908947.3 | 1 | 29903 | 1863425 | 29669 | 99.22 | 4162.81 | 36.5 | 59.9 |
