# Supplementary material for: Rapid genotyping of targeted viral samples using Illumina short-read sequencing data
Source: PLoS One. 2022 Sep 16;17(9):e0274414. doi: 10.1371/journal.pone.0274414 (PMC9481040; doi:10.1371/journal.pone.0274414)
Supplement: S3 Table — (DOCX) [file pone.0274414.s003.docx]

**S3 Table. Detailed statistics as exported with samtools coverage for the HBV dataset.**

| sample_id | rname | startpos | endpos | numreads | covbases | coverage | meandepth | meanbaseq | meanmapq |
| --- | --- | --- | --- | --- | --- | --- | --- | --- | --- |
| SRR12535936 | NC_003977.2 | 1 | 3182 | 249649 | 2752 | 86.49 | 8010.66 | 38 | 59.9 |
| SRR12535937 | NC_003977.2 | 1 | 3182 | 223107 | 2090 | 65.68 | 7357.95 | 37.9 | 59.8 |
| SRR12535946 | NC_003977.2 | 1 | 3182 | 310297 | 3182 | 100 | 9875.27 | 38 | 60 |
| SRR12535938 | NC_003977.2 | 1 | 3182 | 310075 | 3009 | 94.56 | 9586.82 | 37.8 | 59.9 |
| SRR12535947 | NC_003977.2 | 1 | 3182 | 314181 | 3182 | 100 | 9392.32 | 38.1 | 57.8 |
