# Supplementary material for: Rapid genotyping of targeted viral samples using Illumina short-read sequencing data
Source: PLoS One. 2022 Sep 16;17(9):e0274414. doi: 10.1371/journal.pone.0274414 (PMC9481040; doi:10.1371/journal.pone.0274414)
Supplement: S4 Table — (DOCX) [file pone.0274414.s004.docx]

**S4 Table. Detailed statistics as exported with samtools coverage for the RABV dataset.**

| sample_id | rname | startpos | endpos | numreads | covbases | coverage | meandepth | meanbaseq | meanmapq |
| --- | --- | --- | --- | --- | --- | --- | --- | --- | --- |
| SRR12012234 | KT336433.1 | 1 | 11923 | 2675 | 11909 | 99.88 | 15.73 | 36.1 | 59.7 |
| SRR12012235 | KT336433.1 | 1 | 11923 | 79813 | 11916 | 99.94 | 529.40 | 36.1 | 59.9 |
| SRR12012236 | KT336433.1 | 1 | 11923 | 3143 | 11775 | 98.76 | 17.63 | 36 | 59.7 |
| SRR12012237 | KT336433.1 | 1 | 11923 | 144893 | 11923 | 100 | 978.05 | 36 | 59.9 |
| SRR12012238 | KT336433.1 | 1 | 11923 | 106166 | 11915 | 99.93 | 708.35 | 36.1 | 59.9 |
| SRR12012239 | KT336433.1 | 1 | 11923 | 15492 | 11918 | 99.96 | 97.22 | 36.1 | 59.8 |
| SRR12012240 | KT336433.1 | 1 | 11923 | 34641 | 11913 | 99.92 | 222.83 | 36.1 | 59.9 |
| SRR12012241 | KT336433.1 | 1 | 11923 | 34088 | 11912 | 99.91 | 212.80 | 36 | 59.9 |
| SRR12012242 | KT336433.1 | 1 | 11923 | 39417 | 11905 | 99.85 | 246.86 | 36 | 59.9 |
| SRR12012243 | KT336433.1 | 1 | 11923 | 3755 | 11873 | 99.58 | 21.79 | 36 | 59.7 |
| SRR12012244 | KT336433.1 | 1 | 11923 | 1994 | 11685 | 98.00 | 11.206 | 35.9 | 59.5 |
| SRR12012245 | KT336433.1 | 1 | 11923 | 25382 | 11918 | 99.96 | 156.80 | 36.1 | 59.9 |
| SRR12012246 | KT336433.1 | 1 | 11923 | 42129 | 11919 | 99.97 | 276.84 | 36.1 | 59.9 |
| SRR12012247 | KT336433.1 | 1 | 11923 | 13097 | 11905 | 99.85 | 82.51 | 36 | 59.9 |
| SRR12012248 | KT336433.1 | 1 | 11923 | 5038 | 11895 | 99.76 | 29.38 | 36.1 | 59.7 |
| SRR12012249 | KT336433.1 | 1 | 11923 | 10117 | 11911 | 99.90 | 61.76 | 36.1 | 59.8 |
| SRR12012250 | KT336433.1 | 1 | 11923 | 56107 | 11921 | 99.98 | 341.18 | 36 | 59.9 |
| SRR12012251 | KT336433.1 | 1 | 11923 | 18158 | 11912 | 99.91 | 119.40 | 36 | 59.9 |
| SRR12012252 | KT336433.1 | 1 | 11923 | 110577 | 11922 | 99.99 | 671.78 | 36 | 59.6 |
| SRR12012253 | KT336433.1 | 1 | 11923 | 3209 | 11855 | 99.43 | 18.54 | 36 | 59.7 |
| SRR12012254 | KT336433.1 | 1 | 11923 | 17815 | 11914 | 99.92 | 112.33 | 36.1 | 59.9 |
| SRR12012255 | KT336433.1 | 1 | 11923 | 3533 | 11911 | 99.90 | 20.43 | 36.1 | 59.7 |
| SRR12012256 | KT336433.1 | 1 | 11923 | 77218 | 11916 | 99.94 | 497.19 | 36.1 | 59.9 |
