# Supplementary material for: Rapid genotyping of targeted viral samples using Illumina short-read sequencing data
Source: PLoS One. 2022 Sep 16;17(9):e0274414. doi: 10.1371/journal.pone.0274414 (PMC9481040; doi:10.1371/journal.pone.0274414)
Supplement: S5 Table — (DOCX) [file pone.0274414.s005.docx]

**S5 Table. Detailed statistics as exported with samtools coverage for the FCoV sample.**

| sample_id | rname | startpos | endpos | numreads | covbases | coverage | meandepth | meanbaseq | meanmapq |
| --- | --- | --- | --- | --- | --- | --- | --- | --- | --- |
| SRR8352624 | KX722529.1 | 1 | 29174 | 1305 | 27537 | 94.39 | 4.87 | 36.1 | 59.2 |
