# Supplementary material for: Rapid genotyping of targeted viral samples using Illumina short-read sequencing data
Source: PLoS One. 2022 Sep 16;17(9):e0274414. doi: 10.1371/journal.pone.0274414 (PMC9481040; doi:10.1371/journal.pone.0274414)
Supplement: S6 Table — (DOCX) [file pone.0274414.s006.docx]

**S6 Table. Detailed statistics as exported with samtools coverage for the avian Adenovirus sample.**

| sample_id | rname | startpos | endpos | numreads | covbases | coverage | meandepth | meanbaseq | meanmapq |
| --- | --- | --- | --- | --- | --- | --- | --- | --- | --- |
| MT500572 | MG953201.1 | 1 | 45743 | 52014 | 45474 | 99.41 | 121.29 | 33.6 | 58.9 |
