# Supplementary material for: Rapid genotyping of targeted viral samples using Illumina short-read sequencing data
Source: PLoS One. 2022 Sep 16;17(9):e0274414. doi: 10.1371/journal.pone.0274414 (PMC9481040; doi:10.1371/journal.pone.0274414)
Supplement: S7 Table — (DOCX) [file pone.0274414.s007.docx]

**S7 Table. Detailed statistics as exported with samtools coverage for the HSV-1 dataset.**

| sample_id | rname | startpos | endpos | numreads | covbases | coverage | meandepth | meanbaseq | meanmapq |
| --- | --- | --- | --- | --- | --- | --- | --- | --- | --- |
| ERR3316622 | NC_001806.2 | 1 | 152222 | 4948388 | 150538 | 98.89 | 3458.12 | 36 | 55.2 |
| ERR3316623 | NC_001806.2 | 1 | 152222 | 4379968 | 150704 | 99.00 | 2936.14 | 35.3 | 54.3 |
| ERR3316627 | NC_001806.2 | 1 | 152222 | 2819457 | 149421 | 98.15 | 2022.03 | 35.1 | 55 |
| ERR3316619 | NC_001806.2 | 1 | 152222 | 3004641 | 150673 | 98.98 | 2082.84 | 30.8 | 53.9 |
